# Stage 1 embryonal rhabdomyosarcoma of the female genital tract: a retrospective clinical study of nine cases

**DOI:** 10.1186/s12957-017-1110-y

**Published:** 2017-02-07

**Authors:** Guangwen Yuan, Hongwen Yao, Xiaoguang Li, Hongjun Li, Lingying Wu

**Affiliations:** Department of Gynecologic Oncology, Cancer Hospital of Peking Union Medical College and Chinese Academy of Medical Science, No 17 Panjiayuan South Street, Chaoyang District, Beijing, China

**Keywords:** Rhabdomyosarcoma, Gynecologic tumor, Chemotherapy, Surgery

## Abstract

**Background:**

The aim of the study is to investigate the clinical features, treatments, and prognosis of stage 1 embryonal rhabdomyosarcoma of the female genital tract.

**Methods:**

A retrospective analysis was performed on nine cases of stage 1 embryonal rhabdomyosarcoma of the female genital tract. Clinical characteristics, treatments, recurrence, and prognosis were analyzed.

**Results:**

Of the nine patients with embryonal rhabdomyosarcoma, three originated from the vagina and six from the cervix. For the eight patients who initially received surgery, the median survival time was 88 months. As for the six patients that received adjuvant chemotherapy, five of them who received six or more cycles of treatment achieved tumor-free survival and the survival time ranged from 9 to 228 months. The remaining patient, who declined further treatment after two cycles of chemotherapy, relapsed 11 months following the surgery and died 3 months later. Out of the nine patients, only one was initially treated with chemotherapy, and achieved complete remission, but relapsed 21 months later. After a combination of surgery and chemotherapy, this patient remained tumor-free for total of 117 months.

**Conclusions:**

Patients with early stage embryonal rhabdomyosarcoma of the female genital tract have good prognosis, and the combination of surgery and chemotherapy can lead to better outcomes.

## Background

Rhabdomyosarcoma is a rare tumor that occurs mostly in children and adolescents, and rarely in adults, and represents 2–5% of all soft tissue sarcomas [1]. Approximately two-thirds of the cases are diagnosed in children younger than 6 years of age.

Rhabdomyosarcomas (RMS) is divided into three pathological subtypes including embryonal (ERMS), alveolar (ARMS), and pleomorphic rhabdomyosarcomas (PRMS) [2, 3], of which the ERMS and ARMS are the most common subtypes in children and adolescents [1, 4, 5]. PRMS is mainly found in adults with the incidence increasing with age [6].

ERMS mostly occurs in the head and neck regions, whereas PRMS is mostly found in the limbs and trunk [7]. In adults, PRMS is more common than other subtypes [4] and its clinical and biological behaviors are closer to high-grade soft tissue sarcomas. The Intergroup of Rhabdomyosarcoma Study Group (IRSG) founded in 1972 has developed a surgical-pathologic grouping system [8, 9], followed by a pre-treatment clinical staging system [10], and is currently widely used by most scholars.

Rhabdomyosarcoma of the female genital tract is most common in infants, children, and adolescents. Given the rareness of rhabdomyosarcoma of the female genital tract, currently, no standard treatment is available, and recommended strategies are mostly based on treatments for rhabdomyosarcoma of other sites. Here, we performed a retrospective analysis on nine cases of stage 1 embryonal rhabdomyosarcoma of the female genital tract admitted in the Cancer Hospital of Chinese Academy of Medical Science. The clinical manifestations, treatments, and prognosis were analyzed, aiming to provide insights into the treatment and prognosis of this disease.

## Methods

### Study design

A total of nine cases of stage 1 embryonal rhabdomyosarcoma of the female genital tract, admitted in our hospital from January 1975 to December 2014, were analyzed.

The ethics committee of the Cancer Hospital of Chinese Academy of Medical Science approved this retrospective study. All the patients’ records were anonymized and de-identified prior to analysis. Therefore, informed consent was not obtained from the patients.

Medical records of the nine patients were reviewed, and their clinical characteristics, treatments, recurrence, and prognosis were analyzed retrospectively.

All cases with embryonal rhabdomyosarcoma were followed up by phone or mail. The cutoff date of the follow-up was April 1, 2015.

### Statistical analysis

Data were analyzed with SPSS 19.0 statistical software.

## Results

### General clinical features

Of the nine patients, three cases of ERMS primarily occurred in the vagina and six cases occurred in the cervix. The age ranged from 6 to 37 years, and the median age was 21 years. According to the IRSG staging system, all cases were classified as stage 1. In all three cases of rhabdomyosarcoma of the vagina, a vaginal mass was the initial manifestation, and the longest tumor diameter ranged from 1 to 6 cm. According to the 2012 International Federation of Gynecology and Obstetrics staging system, two cases were classified as stage I and 1 case as stage II. Of the six cases of rhabdomyosarcoma of the cervix, five patients initially presented with vaginal bleeding and one presented with increased vaginal discharge. The longest tumor diameter ranged from 0.5 to 12 cm, and according to 2012 FIGO staging system, three cases were classified as stage I, two cases as stage II, and one case as stage III (Table 1).

**Table 1:** Stage, treatment, and prognosis of embryonal rhabdomyosarcoma of the female genital tract

No.	Age (years)	Tumor site	The longest tumor diameter (cm)	Stage (FIGO)	Stage (IRSG)	IRSG group	IRSG risk stratification	Primary treatment	Treatment for recurrence	Status at last follow-up	Survival time^a^ (month)
1	17	Vagina	1.0	I	1	I	Low, subset A	S	C	Dead	25.0
2	6	Vagina	4.9	I	1	III	Low, subset B	C	S+C	NED	117.0
3	34	Vagina	6.0	II	1	I	Low, subset A	S	N	NED	15.0
4	36	Cervix	5.7	IIIA	1	I	Low, subset A	NC+S+C	N	NED	9.0
5	20	Cervix	12.0	IIA2	1	I	Low, subset A	S+C	N	NED	153.0
6	37	Cervix	2.0	IB1	1	I	Low, subset A	S+C^b^	P	Dead	14.0
7	21	Cervix	0.5	IB1	1	I	Low, subset A	S+C	N	NED	151.0
8	21	Cervix	10.5	IIA2	1	I	Low, subset A	S+C	N	NED	228.0
9	23	Cervix	4.5	IB2	1	I	Low, subset A	S+C+R	N	NED	198.0

*IRSG* Intergroup Rhabdomyosarcom Study Group, *S* surgery, *C* chemotherapy, *NC* neoadjuvant chemotherapy, *R* radiotherapy, *P* palliative treatment, *N* without recurrence, *NED* no evidence of disease

^a^The cutoff date of the follow-up was April 1, 2015

^b^Only received two courses of chemotehrapy

### Treatment approaches and prognosis

As of nine patients, eight initially received surgery, and one received just chemotherapy regimen of cyclophosphamide + vincristine + actinomycin for 12 cycles. The patient that received only chemotherapy achieved complete remission but relapsed 21 months after the end of the chemotherapy. After receiving surgery and the postoperative adjuvant chemotherapy, the patient has maintained tumor-free for a total of 117 months so far.

Of the eight patients who initially received surgery with a median survival time of 88 months (9–228 months), six patients received adjuvant chemotherapy, one of whom also received adjuvant external beam radiotherapy. Of the six patients, five patients who completed ≥6 cycles of chemotherapy all survived tumor-free until the cutoff date of the follow-up (survival time range, 9~228 months). The other patient only received two cycles of postoperative chemotherapy (dactinomycin + vincristine + cyclophosphamide) before abandoning treatment voluntarily. Recurrent cancer of the vaginal stump accompanied by brain and lung metastases was found in the patient 11 months after the surgery. The patient then received palliative treatments and died 3 months later, with an overall survival time of 14 months. As for the two patients who did not receive postoperative adjuvant therapies, one has a disease-free survival of 15 months so far, while the other patient had abdominal tumor recurrence 24 months after the surgery. Due to the inoperable condition, the patient received one cycle of chemotherapy and died a month later with an overall survival time of 25 months.

Due to the great time span (40 years) in which these patients received treatments, although all patients were given combination chemotherapies, specific chemotherapy regimens varied. The earliest case received a regimen containing cyclophosphamide and hydroxyurea in 1975. Later, three patients received regimens using triazene melamine, etoposide, or ifosfamide combined with adriamycin and cisplatin, respectively, one patient received paclitaxel, ifosfamide, and cisplatin, and two patients received a regime containing vincristine, actinomycin, and cyclophosphamide.

### Survival time and prognosis

Until the cutoff day of the follow-up, the follow-up duration was from 9 to 228 months, with a median duration of 117 months (range 9~228 months). Nine cases were identified, two were already dead, and the remaining seven were followed up. Seven achieved tumor-free survival with a median survival time of 151 months, two patients died with a survived time of 14 months and 25 months, respectively. The median survival time of the nine patients was 117 months (9~228 months).

## Discussion

The diagnosis of rhabdomyosarcoma mainly depends on the pathological examination of biopsies combined with imaging exams [11]. The 3-year progression-free survival rates of IRSG groups I, II, and III patients are 83, 86, and 73%, respectively (*p* < 0.001) [12], and the long-term survival rate of group IV RMS patients is less than 30% [13, 14]. The 3-year progression-free survival rates of stages 1, 2, and 3 are 86, 80, and 68%, respectively (*p* < 0.001) [12]. Currently, IRSG, now called the Children’s Oncology Group (COG) Soft Tissue Sarcoma Committee, is conducting multiple clinical studies related to RMS.

At present, the multimodal therapy, consisting of chemotherapy and surgery, with or without radiotherapy, has become the recommended treatment for RMS [15]. Using this treatment approach, RMS survival rate has increases from 25% in the 1970s to 71% in the 1990s [8, 10, 12, 16]. Now, COG combines the risk rating and the staging to divide pediatric patients with RMS into low-risk, intermediate-risk, and high-risk groups [8]. The 3-year progression-free survival rates of low-risk, intermediate-risk, and high-risk groups are 88, 55–76, and <30%, respectively. Due to the good prognosis of patients in the low-risk group, studies are currently trying to reduce the intensity of treatment and thereby alleviate treatment-related complications. The survival rate of adolescent and adult patients with RMS (21–56%) is significantly lower than that of children, and several studies are trying to improve its prognosis [15, 17].

The nine patients in this report were all classified as stage 1 according to IRSG staging system, and their median survival time was 117 months (ranged from 9 to 228 months). There were a total of seven cases that achieved disease-free survival, of which five were treated with surgery and postoperative chemotherapy, and one initially received the chemotherapy regimen including vincristine, dactinomycin, and cyclophosphamide, and achieved complete remission, but followed by tumor relapse 21 months later. The patient was then treated with surgery and chemotherapy and has remained tumor-free until now. Another patient, although only received surgery, also has survived tumor-free for 15 months (a shorter follow-up).

As of the two patients who did not survive, one did not receive chemotherapy, and the tumor recurred in the pelvic and abdominal cavities 24 months after the surgery, which caused the death of the patient 1 month later. The other patient only underwent two cycles of postoperative chemotherapy before abandoning treatment voluntarily, and the vaginal stump recurrence and lung metastasis were found 9 months later. The patient was subsequently treated with palliative cares and died 3 months later with an overall survival time of 14 months. Therefore, for female patients with stage 1 ERMS, surgical treatment combined with 6 cycles of adjuvant chemotherapy may be necessary to improve survival, and patients that undergo insufficient postoperative chemotherapy are susceptible to a high risk of recurrence.

In this group of patients, only two were younger than 18 years old, and the rest were over 20 years old, which is inconsistent with the previously reported age distribution of ERMS, possibly due to the majority of pediatric patients being treated in Children’s Hospitals in China. So, most patients admitted at our hospital were adults, resulting in different age distributions.

Specifically for children with RMS, the IRS-III study had used a chemotherapy regimen containing vincristine and dactinomycin (VA), which resulted in 83 and 70% 5-year tumor-free survival rates in the low-risk sub-group A and subgroup B, respectively, while the IRS-IV study had used a chemotherapy regimen containing vincristine, dactinomycin, and cyclophosphamide (VAC), which resulted in 93 and 84% 5-year disease-free survival rates, respectively [10, 12]. Although, the addition of cyclophosphamide improved survival, the occurrence of complications including myelosuppression, infection and infertility, increased [18].

Currently, VAC (vincristine, dactinomycin, and cyclophosphamide) chemotherapy is the standard regimen for children with non-metastatic RMS (moderate-risk or high-risk groups) [19]. A randomized controlled trial (D9803) conducted by COG has compared the efficacy between VAC chemotherapy and the combination of vincristine, topotecan, and cyclophosphamide in patients with moderate-risk RMS, and the results demonstrated 73 and 68% 4-year progression-free survival rates, respectively (*p* = 0.30), indicating that topotecan does not yield superior results over dactinomycin [19].

Since RMS is very rare in adults, selection of chemotherapeutic drugs is mostly based on the outcomes in pediatric patients.

Due to the long time span of this study, the patients received different chemotherapeutic drugs including sarcoma-effective vincristine, doxorubicin, cisplatin, triazene melamine, ifosfamide, and cyclophosphamide. Out of the nine patients, two had received COG recommended VAC (cyclophosphamide, vincristine, and actinomycin). Despite different chemotherapy regimens, those patients that completed more than 6 cycles of chemotherapy showed good prognosis, indicating that in addition to the COG recommended chemotherapy, other drugs may be effective for embryonal rhabdomyosarcoma and worth further investigations. COG is now conducting a number of relevant clinical trials.

The surgical treatments for RMS are site-specific, and tumors should be removed as completely as possible. The level of completeness of the surgical resection directly affects the prognosis. However, if the surgery would cause a significant dysfunction or impact appearance (such as RMS of the head, neck, and genital tract), radical surgery is not recommended, but instead, palliative resection or radiotherapy combined with chemotherapy can be used. In this report, of the three patients with vaginal rhabdomyosarcoma, two patients who underwent surgery both received extended local resection, which resulted in one death with a survival time of 25 months, and one tumor-free survival for 15 months at the end of the study. All six cases of cervical rhabdomyosarcoma received surgery, of whom three underwent hysterectomy. One of these patients, due to the incomplete course of chemotherapy, had recurrence and died later with an overall survival time of 14 months, and the other two survived tumor-free for 151 months and 153 months, respectively. The remaining three patients with cervical rhabdomyosarcoma underwent radical hysterectomy and remained tumor-free for 9, 198, and 228 months, respectively. These results indicate that extended local resection is good enough for vaginal RMS, while for cervical rhabdomyosarcoma, hysterectomy yields better outcomes. Given the young age of these patients, fertility preservation was a major concern. In recent years, patients whose fertility was preserved have been reported [20, 21]. Dehner reported 14 cases with embryonal rhabdomyosarcoma confined to the cervix (aged from 9 months to 32 years) [20], among which 12 patients were found with polypoid tumors, one with cervical nodular masses, and one with a tumor inside the cervical canal. Of the 14 patients, 13 underwent tumorectomy and following the pathological diagnosis of rhabdomyosarcoma, the patients were further treated with a loop electrosurgical excision procedure or cervical conization. For the patient with cervical nodular masses, following tumorectomy, a radical hysterectomy was performed. All patients received postoperative adjuvant chemotherapy. Of the 13 patients who received local surgical treatment, one was lost to follow-up 1 year later and one patient relapsed 1 year after the end of the treatment and was lost to follow-up later. Two patients were found to have pulmonary metastasis 2 and 3 years after the treatment, respectively, but survived tumor-free following local excisions. The remaining eight patients were followed up for 2 to 20 years, and all of them achieved tumor-free survival. The patient who had received radical hysterectomy had recurrence in the pelvic cavity 3 years later but was alive with tumor.

Kriseman analyzed 11 cases of cervical embryonal rhabdomyosarcoma retrospectively [21], among which, according to the FIGO staging system, four were classified as stage IB1, four as stage IB2, one as stage IIA, and two as stage unknown. Of the 11 patients, eight patients underwent cervical conization or cervical tumorectomy, and all patients received postoperative adjuvant chemotherapy (seven cases received VAC, one case received a combination chemotherapy containing doxorubicin, cyclophosphamide, and vincristine). The median follow-up of the patients was 23 months. There were two patients who had recurrence, one of which died of neutropenia following chemotherapy and the other one was alive with tumor (the survival time was not reported). The remaining six patients survived tumor-free for 4–35 months. The authors recommend that for treating patients with an early stage cervical rhabdomyosarcoma, a local excision combined with the postoperative chemotherapy should be considered. However, since the number of cases in these studies is small and the studies are all retrospective, whether or not local excision is an appropriate approach remains to be confirmed.

Radiotherapy is an important component of the comprehensive treatment of pediatric RMS. For patients with tumors that cannot be surgically removed, or with residual tumors or lymph node involvement (IRS groups II, III, and IV), induction chemotherapy followed by concurrent chemoradiotherapy is currently the recommended therapeutic modality [22]. In this report, only one received postoperative adjuvant radiotherapy, which was used in combination with the chemotherapy. The patient has survived for 198 months, which is higher than the median survival time of the five patients who received a combination of surgery and chemotherapy.

## Conclusions

The prognosis of early stage embryonal rhabdomyosarcoma of female genital tract is good, and its standard therapeutic modality is the combination of surgery and chemotherapy. Currently, the standard chemotherapy that IRSG (2001) recommends comprises of vincristine, dactinomycin, and cyclophosphamide [12, 19]. For the surgical treatment of vaginal rhabdomyosarcoma, extended local resection has shown better outcome. For cervical rhabdomyosarcoma, whether fertility-preserving surgical approaches should be adopted requires further investigation.

## Abbreviations

ARMS: Alveolar rhabdomyosarcomas
COG: Children’s Oncology Group
ERMS: Embryonal
IRSG: Intergroup of Rhabdomyosarcoma Study Group
PRMS: Pleomorphic rhabdomyosarcomas
RMS: Rhabdomyosarcomas
